# Risk of depression after Parkinson’s disease, stroke, multiple sclerosis, and migraine in an Iranian population and assess psychometric characteristics of three prevalent depression questionnaires

**DOI:** 10.1016/j.ibneur.2024.01.006

**Published:** 2024-02-03

**Authors:** Mehri Salari, Hossein Pakdaman, Masoud Etemadifar, Fatemeh HojjatiPour, Maede Khalkhali, Nima Mirjamali, Arash Hossein Abadi Farahani

**Affiliations:** a Brain Mapping Research Center, Shahid Beheshti University of Medical Sciences, Tehran, Iran; b Department of Neurology, School of Medicine, Isfahan University of Medical Sciences, Isfahan, Iran; c Student Research Committee, Faculty of Medicine, Shahid Beheshti University of Medical Sciences, Shahid Beheshti Medical University, Tehran, Iran

**Keywords:** Depression, Parkinson Disease, Multiple Sclerosis, Migraine, Stroke

## Abstract

**Objective:**

We aim to evaluate the prevalence of depression in disorders including multiple sclerosis (MS), Parkinson's disease (PD), migraine, and stroke. Also, we detect risk factors for depression occurrence within each disorder. Moreover, we compare the risk factors in these four common neurologic disorders. In advance, we assess the three surveys in order to better comprehend their distinctions.

**Background:**

Depression is a globally prevalent Psychologic disorder and common co-morbidity in neurological diseases. However, it is mostly underdiagnosed in chronic patients and causes numerous adverse effects.

**Methods:**

We used the database of neurology specialty clinics in a hospital in Tehran, the largest city of Iran. Five hundred nineteen patients, including 105 PD patients, 101 patients with stroke, 213 cases with MS, and 100 Migraine patients, were assessed. They were asked about their chief characteristics and disease-specific variables that may cause depression. Moreover, depression criteria were measured with three internationally used scales to study their variances.

**Results:**

Overall, the prevalence of depression in PD, stroke, MS, and migraine, according to the BDI-II scale, were 43.8%, 38.6%, 45.1%, 37.6%, and according to HDRS scale, were 56.2%, 51.5%, 39.4%, and 43.6% respectively. Finally, according to DSM-XC the depression prevalence were 64.8%, 34.7%, 36.2%, and 67.3% respectively. Possible risk factors of depression were lower educational level, disease severity, socioeconomic level, marital or employment status, female gender, higher age, and consumption of some specific drugs.

**Conclusion:**

Depression is a widespread disorder in chronic neurologic conditions. Our data suggests the odds of depression in neurologic disorders depend on the characteristics of the patient and the features of the disease.

## 1 Introduction

Depression (major depressive disorder) presents with low mood, loss of interest in usual activities, and physical symptoms. Major depressive disorder is one of the most common psychologic disorders globally that cause significant public health problems and disturb the quality of life (Peveler, 2002, Kessler and Bromet, 2013). Also, it is stated that depression is the leading cause of disability in the world (Friedrich, 2017). It has become one of the most crucial concerns of governments and families because it increases the risk of suicide and comorbidities and decreases the efforts for treatment (Viguera et al., 2018). Depression can be primary, occurs without any evidence, or it can be secondary, which is preceded by another illness (Costello and Scott, 1991).

Depression is a common co-morbidity in medical disorders such as myocardial infarction. Larsen et al. study showed that one-fifth of patients after three months from MI experience depression (Larsen, 2013). Also, 19% of patients with different types of cancers developed depression, and many experienced subclinical symptoms (Linden et al., 2012). On the other hand, studies comprising neurologic patients similarly presented depression as common co-morbidity (Kanner, 2005, Raskind, 2008). Accordingly, these patients need specific attention. The etiology of this comorbidity has not been fully elucidated. However, depression may occur as a result of affection from the tragic news of the neurologic disorder diagnosis, anatomical or physiological changes due to the situation, the disabilities that come with the disorder, and other unknown etiologies. Moreover, post-stroke depression might be due to lesion location. Depression following PD may share the same etiology with PD or can be a side effect of antiparkinsonian drugs (Kanner, 2005, Raskind, 2008, Knapik et al., 2020). As MacDonalds stated, some areas that cause PD may have a role in depression occurrence, including the ventral tegmental area, hypothalamus, dorsal raphe, and locus coeruleus. Also, right-sided motor symptoms and akinetic rigid PD have a higher rate of depression prevalence (McDonald et al., 2003). Other etiologies, such as inflammatory factors and changes in neurotransmitter levels, have been mentioned (Alba Palé et al., 2017).

As stated, patients with chronic diseases such as neurologic conditions have a great chance of developing depression compared to general population (Knapik et al., 2020). According to M. Kanner’s et al (Kanner, 2005). paper, depression is an independent factor for poor quality of life. So in a situation where depression develops as a comorbidity of another illness, patients need to deal with serious psychiatric problems alongside their chronic conditions. Consequently, their ability to function would significantly decrease, and the whole family's lives will be affected even more (Raskind, 2008). Moreover, depression may delay the response to treatment or change the course and healing process of neurologic problems (Kanner, 2005). As a result, it is concluded that early diagnosis, followed by adequate treatment of depression, reduces the devastating effects of depression and even improves recovery from the primary chronic problem and predicts a better outcome for neurological conditions.

In concurrence with depression and neurologic disorders, depression diagnosis is problematic due to the overlap of the symptoms between medical and psychiatric disorders, especially in MS patients. Therefore, decision-making in this situation is essential for neurologists, and also, they must be skilled in depression features to mark the symptom as psychiatric or neurologic (Kanner, 2005, Raskind, 2008).

Depression is underdiagnosed in several chronic conditions due to numerous reasons. For instance, in low-income populations such as Iran, the patient may not accept that depression is not being a maniac, and they don’t want to be known as a person with mental problems. Thus, they would try to deny this disorder to avoid social stigma. Also, people are even afraid to attend to a psychiatrist due to social disgrace. As enlightened in a Chinese paper by Lao et al (Lao et al., 2016)., it is declared that being illiterate and having a rudimentary knowledge of psychiatric problems will cause being unfamiliar with depression presentations. In addition, elderly patients also tend to avoid psychiatric attendance (Lao et al., 2016, Faisal-Cury et al., 2022).

The overlap of the signs with primary disease leads neurologists to mistake and consider the symptoms for the neurologic disorder. Nevertheless, clinicians shouldn’t neglect this situation (Inagaki et al., 2013). So, it is extremely critical to know how common depression is after prevalent chronic neurological disorders (Viguera et al., 2018, Rickards, 2005).

We aim to assess the prevalence of depression in 4 common neurologic conditions in Iranian populations. According to Abbastabar et al (Abbastabar et al., 2019)., headaches, epilepsy, Alzheimer, Parkinson's disease (PD), and multiple sclerosis (MS) have the highest frequency in Iran, respectively. Consecutively, we chose these common disorders (except epilepsy) to evaluate the risk factors and prevalence of depression in our society. In addition, our aim was to evaluate depression among individuals with chronic neurological disorders, as living with such conditions poses physical and emotional challenges. On the other hand, the discomfort and ongoing physical distress, coupled with a decline in functioning, can significantly impact mood. Social isolation also contributes to the occurrence of depression.

Previous studies predicted the prevalence of depression in various neurological disorders (Bulloch et al., 2015). PD is a progressive neurodegenerative movement disorder and typically causes tremors, stiffness, and slow movements. According to H Rickards et al (Rickards, 2005)**.,** the prevalence of depression in PD patients is probably between 20–45%. Also, depression is the most common mental disorder in MS, a chronic autoimmune disease. The prevalence of depression in MS varies from 25% (Chwastiak et al., 2002) to 50% (Patten et al., 2003, Boeschoten et al., 2017). In addition, according to Luis Ayerbe et al (Ayerbe et al., 2013)., the risk of depression in stroke is about 29% to 52%. Moreover, the chances for depression in people with migraine headaches are 2 to 4 times more than in healthy people (Seng and Seng, 2016, Louter et al., 2016). Additionally, according to Amoozegar et al., the point prevalence of depression in migraine patients is 25% (Amoozegar et al., 2017).

Raskind et al (Raskind, 2008). in a review study estimated post-stroke depression between 20% and 72%. They also stated the prevalence of depression in PD patients ranges from 40% to 50%. In MS patients, depression was present in about 19%− 54%. In another study by Kanner et al (Kanner, 2005)., the range of depression in stroke patients was suggested to be between 30% and 50%. Also, the estimation of depression prevalence in MS was 37%− 54%. 46% was the rate of depression in PD in independent reviews.

Heterogeneity in depression prevalence for each disease is due to using altered methods, different populations and genes, inclusion and exclusion criteria, and other factors like which questionnaires are used to define depression in different studies. Also, it is worth mentioning that even these numbers could be underrated due to the exclusion of patients with communication issues, aphasia, or dementia in the previous studies.

Generally, studies use one scale to detect depression and evaluate the incidence of depression in one neurologic condition. Nonetheless, in this study, we used three different questionnaires to reduce the false positives or false negatives that each questionnaire may have to show the closest result to reality and demonstrate the differences between instruments with the same patients. Also, we estimated the prevalence of depression in four different neurologic disorders to compare the results and evaluate the risk factors of depression in each problem. Furthermore, there hasn’t been any study of depression prevalence and risk factors in Iran society, and this is the first study mentioning patients of an Iranian hospital. A reliable assessment needs global research with different communities, different races, and more patients all around the world (Kessler and Bromet, 2013). Moreover, the risk of depression is dynamic and alters during pandemics, economic circumstances, other aspects of the world, and the country's situation. Thus appraisals need to stay updated in different parts of the world (Moreno-Agostino et al., 2021, Economou et al., 2016). In this cross-sectional study, we used The *Hamilton* Depression Rating Scale (HDRS) and the Beck Depression Inventory (BDI-II) scales which are probably the best-validated tools to quantitatively assess depression (Anderson, 2002). Also, we used the Diagnostic and Statistical Manual of Mental Disorders Self-Rated Level 1 Cross-Cutting Symptom Measure—Adult (DSM XC), a multidimensional instrument spanning many mental health symptoms (Gibbons et al., 2021). Lastly, we measured the prevalence of depression in four common neurologic conditions in the Iranian population and addressed the risk factors of depression in each disorder. These results are helpful for neurologists and psychiatrists to consider this comorbidity and reduce risk factors to evade depression occurrence in neurologic patients.

## 2 Method

### 2.1 Participants

This is a single-center study of all adult patients over 18-year-old patients and were affected with PD, MS, Stroke, and Migraine who were referred to the neurology clinic of the Shohada-e-Tajrish hospital from January 2020 to January 2022. Diagnoses were made by a neurologist based on valid international criteria using accurate clinical neurological exams with para-clinical assessments such as computed tomography (CT) or magnetic resonance imaging (MRI) brain scans. Our final sample exclude Patients with indeterminate diagnosis, and patient unable or reject participation.

This study was approved by the Ethics committee of Shahid Behehshti University of Medical Sciences. Ethic number is IR.SBMU.RETECH.REC.1399.745.

### 2.2 Assessment

The data gathered by interviewing with patients who were intended to the neurology clinic. Through this survey, the patient’s characteristics such as age, educational level, living status (categorized as alone, with family, with nurses) marital status (classified as either currently married or not), employment status, sociodemographic level, alcohol consumption, smoking, drug abuse, and physical activity were assessed by medical students. Patients were asked about clinical data, including past medical and drug history, and cognitive function, with a Mini-mental state exam (MMSE). Also, specific variables of each disease, such as duration of the condition, age of onset and symptoms were studied. Additionally, patients underwent a disability evaluation. The Expanded Disability Status Scale (EDSS) for MS, The Hoehn and Yahr scale (H&Y) for PD, The Modified Rankin Score (MRS) for stroke and The Migraine Disability Assessment (MIDAS) for migraine.

Finally, we used three different questionnaires to estimate depression including, BDI-II, HDRS, and DSM XC. The BDI-II survey is a self-reported questionnaire that contains 21 items on a 4-point scale from 0 (symptom absent) to 3 (severe symptoms), and a minimum of 13 is needed to diagnose a patient with depression (Jackson-Koku, 2016). This instrument was asked by a clinicians and been answered by the patient. This survey is mentioned to be quietly valid and reliable in Persian language (Ghassemzadeh et al., 2005, Dadfar and Kalibatseva, 2016, Ahmadi et al., 2019). Another instrument that was used to diagnose depression was the HDRS, which is a semi-structured interview and measured by a trained clinician. This oldest, most widely used and validated instrument, (Anderson, 2002) has 17 items each item is scored on a scale of 0 (not present) to 4 (severe). On this scale, diagnosis of depression was based on reaching the minimum score of 8 (Sharp, 2015). The last questionnaire that was also completed by clinicians is DSM-XC scale, that comprise 23 questions on a 4-point scale from 0 (none) to 4 (nearly every day). The APA recommended thresholds for DSM XC used to diagnose depression (Mahoney et al., 2020).

As mentioned earlier, depression diagnosis in the presence of other neurologic disorders is challenging for clinicians. Therefore, we used three different instruments to avoid an overlooked depression in a scale and bias in a questionnaire. Also, according to McDonalds et al (McDonald et al., 2003)., using self-rated BDI, which has high sensitivity and specificity, is more reliable for a distinction between depression and similar symptoms of neurologic disorders (Anderson, 2002). It is worth mentioning that using one particular scale in depression research may lead to idiosyncratic results and threaten the validity of a very large and important field of research**.** On the other hand, since most studies only use one questionnaire, it is somewhat incomparable with other research projects. This is where combining three surveys is useful when comparing the findings with those of other studies.

We included a total of 519 patients with chronic neurologic diseases, including MS, PD, migraine, and stroke.

105 PD patients were included. Specific variables of the disease include drinking tea or coffee, age of onset, duration of the disease, the first sign of the disease, first affected side of the patient, dominant symptom, having a freezing gate or not, and whether using DBS were evaluated. Subjects not meeting the Brain Bank diagnostic criteria (Hughes et al., 1992) for PD, patients with other movement disorders that may present similar to PD, such as atypical parkinsonism, people suffering from major medical disorders that interfere with the patient’s ability to communicate, and patients with cognitive impairment (MMSE<10) were excluded. Other patients who were suffering from PD and meet the inclusion criteria were included. Disability of Parkinson patients assessed by The Hoehn and Yahr scale (H&Y) (Martinez-Martin et al., 2018).

Also, 101 ischemic stroke patients confirmed with MRI or CT scan were included. Specific variables of the disease include where in the brain did the strokes take place, the number of attacks, the age of the first attack, the first affected side of the body, and whether using rehabilitation were also assessed. Alternative diagnoses, subjects who suffer from severe aphasia or dysarthria or are old enough to cause communication problems, severe cognitive impairment (MMSE<10), and any problem that harms evaluation (e.g. not cooperating), were excluded. Patients diagnosed with other conditions, even severe illnesses, who meet the inclusion criteria were included. For stroke patients, we adopted The Modified Rankin Score (MRS)35 to indicate the disability.

Inclusion criteria for 213 MS patients were confirmed MS diagnosis based on Mcdonald criteria (Thompson et al., 2018), and ability to answer the questions appropriately. Also, patients with coexistence of other mental or physical disorders were included. The exclusion criteria were like previously mentioned disorders. The specific variables of the disease that were studied include age of onset, duration of the disease, type of MS, and last attack. The *Expanded Disability Status Scale* (EDSS) (Meyer-Moock et al., 2014) was used to measure disability in MS patients.

Lastly, 100 patients at least 18 years of age who suffered from any type of migraine, according to the International Classification of Headache Disorders (ICHD) (IHS et al., 2018), who were able to participate in the study were included. Patients who were unable (MMSE<10) or rejected participation were excluded. Specific variables of the disease include the age of onset, duration of the disease, having MRI signs, and characteristics of migraine ( pulsatile, intensity of pain, number and duration of attacks, worsening with activity, aura, photophobia, nausea vomiting, the origin of the pain, being one or two-sided, and stimulants) were questioned. The Migraine Disability Assessment (MIDAS) (Edmeads et al., 2001) score was applied for disability of patients affected with migraine.

Individuals were all Iranian or Afghan and characteristics of patients are summarized in Table 1.

**Table 1:** Patient Characteristics by Center.

		total	stroke	PD	MS	Migraine	p value
Number (%)		519	101(19.4)	105(20.2)	213(41)	100(19.4)	
FEMALE (%)		295(56.7)	33(11.2)	37(12.5)	157(53.2)	68(23.1)	P < .001^a,b,c,d,e^
age(years) mean± SD			65.42 ± 11.5	57.01 ± 12.1	36.52 ± 10.03	38.12 ± 13.8	P * *< .001^a,b,c,d,e,f^
EDUCATION (%)	illiterate		21.8	8.6	0.5	2.0	
	elementary		31.7	9.5	2.8	0.0	
	middle school		1.0	1.0	1.9	2.0	
	high school		13.9	6.7	8.0	5.9	
	diploma		19.8	40.0	39.9	38.6	
	bachelor		8.9	14.3	35.2	29.7	
	master		1.0	9.5	10.3	17.8	
	doctorate		2.0	1.9	1.4	4.0	
married (%)		404(77.7%)	96(95)	90(85.7)	156(73.2)	63(61.4)	
Duration(month) mean± SD				97.38 ± 87.4	97.84 ± 89.1	198.48 ± 152.4	P * *< .001^b,d^
job (%)		186(35.8)	20(20)	29(27.6)	80(37.5)	57(56.4)	
alcohol consumption^1^ (%)		33(6)	6(6)	7(7)	9(4)	11(11)	
MMSE mean ± SD		27.8(4.2)	23.53(5.6)	25.75(3.8)	28.7(2.1)	29.54(1.5)	

SD=standard deviation, PD= Parkinson disease, MS= multiple sclerosis

a= MS significantly different from Parkinson, b= ms significantly different from migraine, c=ms significantly different from stoke, d=migraine significantly different from parkinson, e=migraine significantly different from stroke, f= Stroke significantly different from Parkinson.

Alcohol consumption referred to patients who drink regularly (more than one a week)

### 2.3 Statistical analyses

Statistical analyses with values with 95% confidence intervals were calculated using IBM SPSS Statistics. Descriptive statistics were used to summarize patient characteristics for each specific disease regarding age, gender (male vs. female), education (illiterate, elementary, middle school, high school, diploma, bachelor, master, doctorate), marital status (married vs. not married) socioeconomic level (below standard, standard, and above standard), job (with or without a job at the time), using alcohol or tobacco, hours of activity, living status (family, with a nurse, alone), psychologic history ( with or without psychologic history) disease-specific performance scale (MRS, EDSS, H&Y), and cognitive function (MMSE).

By means of chi-square, Mann-Whitney, and Kruskal-Wallis tests we drew comparisons between all the variables related to four disorders while considering three instruments and we analyzed variables among four diseases individually.

Univariate logistic regression models were used to identify patient characteristics association with depression within disorders. Also, we made multiple logistic regression models including all mentioned predictors and patients. Finally, all associations significant at P < 0.05 were displayed.

## 3 Result

We examined records of 105 patients diagnosed with PD (35.2% female and 64.8% male, and the general mean age was 57.01 years). Moreover, we examined 101 individuals with stroke (32.7% female and 67.3% male with an overall mean age of 65.42 years) and 213 participants suffering from MS (73.7% female and 26.3% male with a mean age of 36.52 years. Also, 100 individuals affected by migraine (67.3% female and 32.7% male with a mean age of 38.12 years) were assessed. The information concerning all patients is shown in Table 1. We intended to evaluate prevalence of depression in neurologic disorders and find the variables role concerning depression in the four prevalent neurologic conditions.

**Fig. 1:**
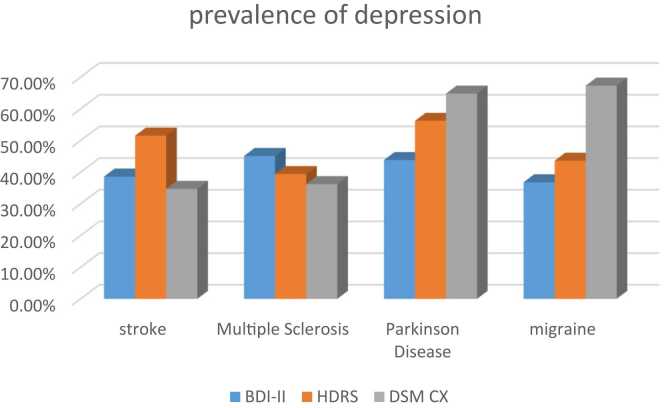
Prevalence of depression within four disorders according to three questionnaires.

Our primary outcome was depression Prevalence: the point prevalence of depression estimated in PD, stroke, MS, and migraine were 43.8%,38.6%, 45.1%, 38.6% respectively according to BDI-II and were 56.2%, 51.5%, 39.4%, 43.6%, respectively according to HDRS and were 64.8%, 34.7%, 36.2%, 67.3% respectively according to DSM-XC Fig. 1.

As we can see, contrary to what we had anticipated, the point prevalence of depression in an illness varies across different scales. The fact that questionnaires concentrate on various depressive presentations may account for this discrepancy. According to Santor et al (Santor et al., 2006)., we can observe an excessive variability of item content in the most commonly used depression severity assessments. The potential for issues to arise increases with scale heterogeneity. For example, the HRSD has a considerably higher proportion of somatic items, which are simpler to evaluate on a clinician graded scale, whereas the BDI covers more cognitive questions (Fried, 2017). The third factor contributing to the diverse point incidence of depression for a given disease is that each instrument was developed using various clinical viewpoints relating what depression is. The BDI is based on the concept of Beck and his colleagues about depression, which is in accordance with Beck's theory and covers many cognitive symptoms, but on the other hand, each questionnaire were made for different purposes so their results may be in dissimilarity, which is another reason for the heterogeneous point prevalence of depression for one disorder. For example The HRSD was created to evaluate the severity of symptoms in patients who already had an MDD diagnosis (Fried, 2017). Additionally, many popular scales have different categories for grading the severity of depression in patients. While scales are often multidimensional and evaluate a variety of concepts, these views are not universal among scales. Consequently, adopting alternative metrics could lead to different outcomes. Last but not least, Fried et al (Fried, 2017). indicate that the average overlap between all scales is 0.36, indicating a weak similarity between the 7 scales that contain HDRS and BDI. The outcome for the same group of depressed people may then vary.

It is important to note that the psychometric properties of the three global measures appear to differ along distinct axes fully addressed in the discussion chapter.

***Univariate analysis for Each Disease**:* we calculated the effect of each factor within each disease which is shown in Table 2. Mean age, duration, and gender were significantly different between the disorders.

**Table 2:** univariate analysis for each disorder.

	stroke			PD			MS			migraine		
p value	BDI-II	HDRS	DSM-XC	BDI-II	HDRS	DSM-XC	BDI-II	HDRS	DSM-XC	BDI-II	HDRS	DSM-XC
Age	< .05	.121	.527									
Marriage										< .05	.215	< .05
Gender	< .05	< .001	.112							.29	< .05	.56
Duration				< .05	.72	.76						
Disabilities	< .001	< .001	< .001				.265	< .001	< .05	.792	< .05	.102
Education	< .05	< .05	< .05	< .05	< .05	< .05	< .001	< .05	< .05	.52	.64	< .05
Socio-econimic level	< .05	.407	.159				< .05	.117	.134			
Job	< .05	< .05	.124				< .05	< .05	.981			
Alcohol consumption							.15	.103	< .05			
Previous attack	< .05	.829	.624									
Antipsychotic drugs							.583	.061	< .05			
SSRI										.496	< .05	.999
Digitals	< 0.05	0.117	0.657									
Benzodiazepin	0.069	< 0.05	0.365									
Opioid	0.157	0.123	< 0.05									

In PD patient according to BDI-II duration of the disease and according to all three scales lower education were significant predictors of depression.

In MS patients, according to BDI-II, lower education, worse socioeconomic level, and unemployment, according to DSM-XC, higher EDSS, lower education, alcohol abuse, and antipsychotic drugs, and according to HDRS scale, higher EDSS, lower education, and joblessness had associations with depression.

In stroke patients, according to BDI-II, aging, female gender, higher MRS, lower education, worse socioeconomic level, unemployment, history of stroke, using digitals, and according to DSM-XC higher MRS, lower education, using opioids, and according to HDRS scale female gender, higher MRS, lower education, unemployment, and using benzodiazepine correlates with depression.

In patients with migraine, according to BDI-II, lower education, and being single, according to DSM-XC lower education, and being single and according to HDRS female gender, higher MIDAS, and using antipsychotic drugs had correlations with depression.

***Combined disease univariate analysis:*** not having a job according to HDRS and BDI-II scale and lower educational level according to BDI-II scale increase the chance of depression in all disorders.

***The univariate logistic regression***: In a logistic regression analysis with BDI-II as the dependent variable, not having a job had a univariate association with depression, and using HDRS as a dependent variable, not having a job, severity, higher age of onset and lower educational level were associated with depression. And with DSM XC as the independent variable, severity and higher smoke pack-year showed univariate association with depression. (Table 3).

**Table 3:** Univariate logistic regression.

		job	age	education	ageof onset	smoke pack year	severity
BDI-II	b coefficient	-0.095					
	p value	0.032					
HDRS	b coefficient	-0.115	0.118	-0.151	0.108		0.91
	p value	< 0.001	< 0.05	< 0.001	< 0.05		< 0.05
DSM XC	b coefficient					0.143	0.21
	p value					< 0.001	< 0.001

***Combined Disease Multiple Regression Model***: multiple models included age, sex, education, marital status, socioeconomic level, job (whether working or not), smoke, alcohol consumption, MMSE, age of onset, and disease severity. Smoke pack-year, according to all three scales, had significant connection with depression. According to the HDRS scale, age, job, and severity of the disease had significant relationship with depression. And according to DSM XC, education and disease severity increase the risk of depression in accordance with other variables. (Table 4).

**Table 4:** multiple regression model.

		job	age	education	smoke pack year	severity
BDI-II	b coefficient				0.144	
	p value				< 0.05	
HDRS	b coefficient	-0.116	-0.57		0.128	0.16
	p value	< 0.05	< 0.05		< 0.05	< 0.05
DSM XC	b coefficient			-0.132	0.193	0.254
	p value			< 0.05	< 0.001	< 0.001

## 4 Discussion

**This study was one of the first to predict depression prevalence in Iran with three different scales in patients with common chronic neurologic conditions include stroke, MS, PD, and migraine. Also, we assessed the variables that associates with depression in 4 neurologic disorders in Iranian patients. According to BDI-II, HDRS, DSM XC scales depression prevalence in stroke patients were 38.6%, 51.5%, 34.7% in MS patients were 45.1%, 39.4%, 36.2% in PD patients were 43.8%, 56.2%, 64.8%, in migraine patients were 36.8%, 43.6%, 67.3% respectively which is slightly higher than the earlier report of depression incidence in the Iranian patients (**Tahan et al., 2020**). Moreover, these results were similar to previous reports of depression in these four neurologic disorders in the world (**Rickards, 2005; Bulloch et al., 2015; Chwastiak et al., 2002; Patten et al., 2003; Ayerbe et al., 2013; Seng and Seng, 2016). **Prevalence variation in a disorder attributed to rating scales, populations, inclusion and exclusion criteria, and many other etiologies.**

There are copious studies that evaluate the prevalence of depression in a neurologic disorder or assess the risk factors of depression in a condition. However, this study is the first study in Iranian population that used three different scales for primary measures of depression and compared four frequent neurologic conditions concurrently. Also, we evaluate association factors of depression in each disease. Furthermore, we assess relation between variables and depression within all disorders. Accordingly, results are comparable for scales and disorders. It’s worth mentioning that the results are more accurate due to using the most common tests together in a single population. Moreover, data collection was made by medical students not the doctors themselves to avoid observing bias, and the data are from one hospital-based cases in one race.

Viguera et al (Viguera et al., 2018). study evaluated the prevalence of depression with Patient Health Questionnaire-9 (PHQ-9) among a large sample of patients who were referred to a tertiary care hospital with Stroke, Epilepsy, and MS. Prevalence of depression in stroke, Epilepsy, and MS were 23.0%, 33.1%, 29.2% respectively. They concluded in stroke patients, lower age, female sex, and lower income increased the odds of depression. Meanwhile, we had an association between higher age and depression, and other associations repeated in our study. Moreover, they concluded in MS patients, lower age, unmarried status, lower-income, and higher PS (worse performance) are associated with depression. Nonetheless, we didn’t find any connection between lower age and unmarried status in MS patients with depression. In Viguera's study, as in this study, increasing disease severity was an independent forecaster of depression across all conditions.

Andrzej et al., in a cross-sectional study on MS, stroke, and Parkinson's, patients showed that there are no associations between patient’s age, duration of a condition, or concomitant diseases with depression in these disorders (Knapik et al., 2020). We also couldn’t find any association between other medical situations and depression, though there were some relationships between odds of depression with age or duration of the disease. As Gordon parker (Parker and Brotchie, 2010) suggested we found a relation between female gender and depression in migraine and stroke patients.

In our study, higher education reduces the chance of depression in all four disorders. It has been mentioned before that the level of education and depression have an inverse relationship (Shen, 2020, Kranjac et al., 2017). Besides, in this study, we observe that some drugs correlate with higher odds of depression. In stroke patients, digoxin, benzodiazepine, opioid usage, and antipsychotics in MS patients showed significant association with depression. Drug-induced depression has been well studied earlier and showed that there are relations between digoxin and depression, (Patten and Love, 1997) antipsychotics and depression, (Ganzini et al., 1993) also opioid usage and depression (Scherrer et al., 2017).

We couldn’t find any association between other illnesses and odds of depression in stroke patients, as stated in Lingru Wang et al (Wang et al., 2016). article. However, according to Jorgensen et al (Jørgensen et al., 2016)., diabetes, and as reported by Jiang et al (Jiang et al., 2014)., hypertension has a significant chance of causing depression in stroke patients (Jørgensen et al., 2016). Single cohabitation status was also mentioned as a risk factor for depression by Wang et al (Wang et al., 2016)., and Jorgensen et al (Jørgensen et al., 2016)., which wasn’t significant in this study. In Jiang et al (Jiang et al., 2014)., study socioeconomic level, previous stroke, and according to Joergensen et al (Jørgensen et al., 2016). older age, female gender, and basic educational attainment, and according to Tsai et al (Tsai et al., 2016)., female gender were significant risk factors of depression, similar to our result. Nonetheless, family history or personal history of depression wasn’t significant in this study though it is mentioned by Morris et al (Morris et al., 1992)., Perrain et al (Perrain et al., 2021)., and Jorgensen et al (Jørgensen et al., 2016).

We found expected association between severity of the MS and depression with the EDSS scale and no significant association between depression and age or sex, as revealed earlier by Solaro et al. article (Solaro et al., 2016). Although other studies support the fact that sex isn’t a risk factor for depression found a higher chance of depression with younger age (Patten et al., 2000, Beal et al., 2007).

The severity of the disease in Parkinson patient’s associated with depression occurrence, as suggested in previous studies (Giladi et al., 2000, Leentjens et al., 2013, Tandberg et al., 1997). Worse cognitive status and worse functioning have been also mentioned as risk factors for depression in PD patients (Leentjens et al., 2013, Tandberg et al., 1997). Moreover, according to Leentjens et al., female sex, history of depression, or family history of depression have a significant association with depression. Whereas none of the above variables have been significant cofactors for depression in our study, the only similar association was the duration of the disease, which is mentioned in Leentjens et al (Leentjens et al., 2013)., Tandberg et al (Tandberg et al., 1997)., and Guze et al (Guze and Barrio, 1991). studies. Also there was no link between depression and dominant symptom of Parkinson in our study though Guze and Barrio (Guze and Barrio, 1991) indicated that in patients with greater bradykinesia and rigidity depression is more common.

There are much fewer studies about depression risk factors in migraine patients. In Pearl et al. study (Pearl et al., 2020), severe depression showed correlations with higher MIDAS scores and activity impairment.

The diversity between studies is possibly due to variances in sample sizes, analysis, population races, limitations of studies, questionnaires, the social and economic situation of the countries, and biases. As a result, other vast studies are needed to confirm these results in different parts of the world. Furthermore, till now, there is no unified pathophysiology of this high prevalence of depression in neurologic conditions, so studies are needed to elucidate the mechanism of depression in neurologic disorders. Though the exact etiology of depression in neurologic conditions hasn’t been specified, there are theories on each problem. villa et al (Villa et al., 2018). suggested that post-stroke depression might be a consequence of neuroinflammation, reciprocal modulation of neurotransmitter systems, neuroendocrine activation, neuronal plasticity, and vascular factors. In Parkinson disease, Guze and Barrio (Guze and Barrio, 1991) stated that depression occurrence might be attributed to fluctuations in dopamine and serotonin levels. According to a systematic review by Alba Pale et al (Alba Palé et al., 2017)., depression in MS patients may be induced by inflammatory factors that damage the brain and reduce serotonin levels. Also, Zhang et al (Zhang et al., 2019). showed in a systematic review that migraine and depression have a bidirectional relationship, and the cause of this situation poorly understood and needs further research. However, this comorbidity might be based on genes influence or the existence of pain in each problem.

Depression diagnosis is challenging in patients with neurologic conditions because their symptoms mimic depression, and there are no specific criteria that can distinguish psychiatric and medical analogous symptoms. Until new guidance in this manner discovers, neurologists should consider prescribing antidepressants.

This study's findings indicated that different questionnaires yielded varying percentages of depression within a same disease. To discuss the features of each survey according to Simeng Ma et al (Ma et al., 2021). the test-retest consistency of HDRS is poor, for items including loss of insight, genital symptoms, hypochondriasis and weight loss. Also, on this assessment anxiety symptoms and adverse effects of drugs are measured besides depression features. However, when the degree of depression is higher, the higher score is not selected for several items. Furthermore, even in the most severe case of depression, there is little chance of selecting the response with the highest score for certain parts, such as emotions of guilt, retardation, mental anxiety, physical anxiety, and hypochondriasis. These results imply that the severity of depression will not be a determining factor in selecting a higher score. Besides, the loss of insight item yielded inconsistent results and provided insufficient information for assessing the severity of depression (Bagby et al., 2004). The sexual activity item may also have a negative effect on the overall depression score because, in Iran, discussing one's sexual life is considered a social stigma, and people tend to avoid discussing it in public. In conclusion the rating scheme for more items are problematic therefor, the HRDS's capacity to detect changes is low (Bagby et al., 2004). It is worth mentioning that the worthlessness and concentration difficulties are not included individually.

For BDI survey it is mentioned that this questionnaire overestimates the severity of depression. On the other hand, it’s a self- reported questionnaire, consequently, patients may exaggerate or conceal their symptoms and cause data loss (Hajduska-Dér et al., 2022). other disadvantages that were mentioned by ritcher et al (Richter et al., 1998). are high item difficulty, unstable scores over short time intervals (over the course of a day), low discriminant validity against anxiety, no representative standards, and thus questionable objectivity of interpretation. Lack of specificity, sensitivity to slight mood swings, and demand characteristics that confound results are other flaws (Lubliner, 2011)**.**

Subsequently, using BDI alone is inadequate for nosologic depression diagnosis, in other words this questionnaire was designed to diagnose syndromic depression. Hence, using BDI with other measurements offer a better diagnostic results.

Regarding DSM XC, its sensitivity mentioned to be low and some items had inadequate validity and some were situationally positive. As a result their score is dependent upon the additional factors (Mahoney et al., 2020).

There has been some limitation in our study that needs to be mentioned. Our population is from a single hospital, and all are Iranian except one afghan. Diagnoses of neurologic disorders were made by a single neurologist, which may cause biases. Measuring socioeconomic level and activity per day was not according to a universal scale, so we were unable to analyze the data. Another limitation of this study was the subjective interview of the patient’s background characteristics which may be overlooked or forgotten by the patient. Also, the drug history of MS and PD patients had many missing data. In disease selection, although epilepsy is mentioned as the second most frequent neurologic disorder in Iran, this disorder did not include in this study due to the lack of data of neurology specialty clinics in our hospital. The populations are not matched, and the last limitation is that the depression before and after the neurologic disorder symptoms begin isn’t separated from each other.

## 5 Conclusion

It is accepted that the prevalence of depression in neurologic conditions such as stroke is higher than in the normal population.

In this study, we confirm this theory with three questionnaires in an Iranian population. Besides, the association of factors and depression within each disorder was assessed. Also, risk factors of depression in all four neurologic conditions were studied. This article indicates that education, gender, age, job, disabilities, alcohol consumption, specific drugs, socioeconomic level, marriage status, smoking, and duration of the disease play a role in depression incidence. Moreover, as the general population age and neurologic tools advancement occur, the number of known neurologic patient increase. Therefore, the comorbidities such as depression become a great concern. This national problem needs more attention. Moreover, further research is needed to prevent this burden in chronic neurologic situations.

## Ethics approval

The questionnaire and methodology for this study was approved by the Human Research Ethics committee of the Shahid Beheshti University of Medical Sciences (IR.SBMU.RETECH.REC.1399.745).

## Funding

This research did not receive any specific grant from funding agencies in the public, commercial, or not-for-profit sectors.

## CRediT authorship contribution statement

Conceptualization: Mehri Salari, Hossein Pakdaman, Masoud Etemadifar; Methodology: Mehri Salari, Hossein Pakdaman, Masoud Etemadifar; Software: Fatemeh Hojjati Pour; Formal analysis: Fatemeh Hojjato Pour; Investigation: Fatemeh HojjatiPour, Maede Khalkhali, Nima Mirjamali, Arash Hossein Abadi Farahani; Resources: none; Data Curation: Fatemeh Hojjati Pour; Writing - Original Draft: Fatemeh Hojjati Pour; Writing - review and editing: Mehri Salari; Visualization: Fatemeh Hojjati Pour; Supervision: Mehri Salari, Hossein Pakdaman, Masoud Etemadifar; Project administration: Mehri Salari, Hossein Pakdaman, Masoud Etemadifar; Funding acquisition: none.

## Declarations of interest

None.
